# Phenotypic characterization of Class III malocclusion using three-dimensional analysis on a sample of Yemeni population: a retrospective cross-sectional study

**DOI:** 10.1590/2177-6709.30.3.e2524129.oar

**Published:** 2025-09-19

**Authors:** Farida BUKAREZA, Ghamdan AL-HARAZI, Sarah AL-RAI

**Affiliations:** 1 Sana’a University, Faculty of Dentistry, Orthodontics department (Sana’a, Yemen).; 2 Saba University, Faculty of Dentistry, Conservative, Preventive and Orthodontics department (Sana’a, Yemen).

**Keywords:** Angle Class III malocclusion, Principal component analysis, Cluster analysis, Cone beam computed tomography, Má oclusão de Classe III de Angle, Análise de componentes principais, Análise de *clusters*, Tomografia computadorizada de feixe cônico

## Abstract

**Introduction:**

Class III malocclusion is one of the abnormalities in the craniofacial development that can be affected mainly by genetic and environmental components. It is a clinical challenge, due to limited understanding of its etiology. Exploring its prototypical diversity helps to identify its etiological details.

**Objective:**

This study aimed to characterize Class III malocclusion subgroups depending on the phenotypic characteristics, by using cone beam computed tomography (CBCT) in a selected group of Yemeni subjects.

**Methods:**

A retrospective cross-sectional study was performed using 80 pretreatment CBCT of patients (46 males, 34 females), with ages of ≥18 years for males and ≥16 years for females. All cases had Class III malocclusion ranging from mild to severe. The total of 74 measurements were three-dimensionally analyzed using Invivo^®^ 6.0 software. These measurements were categorized into 46 skeletal, 18 dentoalveolar, and 10 soft tissue variables. Principal component analysis (PCA) and cluster analysis (CA) were performed to identify the most common clusters in skeletal Class III malocclusion phenotypes.

**Results:**

The PCA revealed 8 axis models, which were responsible for 78.9% of the variation of the data produced from the 74 variables. The first four components accounted for 56% of the total variations, explained mainly the sagittal, dental, vertical, and anteroposterior relationships in the data. The CA revealed four skeletal Class III malocclusion phenotypes: C1=32.8%; C2=28.4%; C3=28.4%; and C4=10.4%.

**Conclusion:**

Based on CBCT, four phenotypes of skeletal Class III malocclusion were identified among Yemeni population. These findings help to provide differential diagnosis that lead to set up an accurate and effective treatment plan.

## RESUMO

**Introdução:**

A má oclusão de Classe III é uma das anomalias no desenvolvimento craniofacial que pode ser afetada principalmente por componentes genéticos e ambientais. Constitui um desafio clínico, devido à compreensão limitada de sua etiologia. Explorar sua diversidade prototípica pode ajudar a identificar seus detalhes etiológicos.

**Objetivo:**

O objetivo deste estudo foi caracterizar os subgrupos da má oclusão de Classe III, dependendo das características fenotípicas, usando a tomografia computadorizada de feixe cônico (TCFC) em um grupo selecionado de indivíduos do Iêmen.

**Métodos:**

Foi realizado um estudo transversal retrospectivo usando TCFCs pré-tratamento de 80 pacientes (46 homens, 34 mulheres) com idades de ≥18 anos para homens e ≥16 anos para mulheres. Todos os pacientes tinham má oclusão de Classe III, variando de leve a grave. No total, 74 medidas foram analisadas tridimensionalmente com o *software* Invivo® 6.0. Essas medidas foram categorizadas em 46 variáveis esqueléticas, 18 dentoalveolares e 10 de tecidos moles. A análise de componentes principais (ACP) e a análise de *clusters* (AC) foram realizadas para identificar os agrupamentos mais comuns nos fenótipos esqueléticos da má oclusão de Classe III.

**Resultados:**

A ACP revelou 8 modelos de eixos, que foram responsáveis por 78,9% da variação dos dados produzidos a partir das 74 variáveis. Os quatro primeiros componentes foram responsáveis por 56% das variações totais e explicaram principalmente as relações sagital, dentária, vertical e anteroposterior nos dados. A AC revelou quatro fenótipos esqueléticos de má oclusão de Classe III: C1=32,8%; C2=28,4%; C3=28,4%; e C4=10,4%.

**Conclusões:**

Com base nas TCFCs, foram identificados quatro fenótipos de má oclusão esquelética de Classe III na população do Iêmen. Esses achados ajudam a fornecer um diagnóstico diferencial que leva ao estabelecimento de um plano de tratamento mais preciso e eficaz.

## INTRODUCTION

Treatment of Class III malocclusion is a challenging task for orthodontists.^1^ The prevalence of Class III malocclusion is low and varies depending on ethnic background.^2^ The prevalence of Class III malocclusion among Yemeni adolescents was 13.5% for incisor relationship and 2.5% for bilateral canine relationship.^3^ Class III malocclusion is affected by wide ranges of congenital and environmental factors.^4^ An accurate diagnosis and prognosis of Class III malocclusion is essential for its management. A normal occlusion and improved facial esthetics for skeletal Class III patients can be achieved by growth modification, orthodontic camouflage, or orthognathic surgery. The treatment strategies can be based on dental development and skeletal development or after growth cessation.^5^

In Dentistry field, cone beam computed tomography (CBCT) is an accurate diagnostic tool for assessing soft and hard tissues, defining treatment plans, and evaluating their outcomes.^6^ ^,^ ^7^ According to the literature, CBCT image has a high degree of measurement accuracy in all three planes of space.^8^ For example, a study using Björk and Jabarak cephalometric analysis^9^ on CBCT lateral cephalograms in adults with different sagittal skeletal malocclusions concluded that skeletal Class III malocclusion was strongly differentiated from the other sagittal Classes, particularly in the mandibular aspect.^9^ Different previous studies used multivariate analysis, such as principal component analysis (PCA) and cluster analysis (CA), to aid further detailed information about the characterization of Class III phenotypes.^10-12^ PCA is a dominant approach that reduces complex data into data that is easier to interpret. CA is a multivariate technique that classifies patients according to the levels of similarity. CA can be used to show distinct patterns of correlation among phenotypic variables, allowing the orthodontist to develop accurate treatment planning to address the underlying skeletal problem in Class III patients. Several studies stated that cluster analysis can better characterize the subtypes of other diseases^10^ ^-^ ^12^; therefore, CA has been used to find the subgroups of Class III malocclusion and then identify the severity between subgroups.^10^ ^,^ ^11^

Studies on Class III phenotypes revealed various sub-phenotypes among different studies and populations, ranging from fourteen^15^, seven^13^, nine^14^, and four^10^ ^,^ ^11^ different sub-phenotypes in Asian populations to five clinically distinguishable phenotypes among Caucasian individuals,^12^ ^,^ ^16^ and six homogeneous sub-phenotypes in southern European subjects.^17^

Three-dimensional analysis probably is the best way to approach the phenotypes of Class III malocclusion.^11^ Up to our knowledge, there is no study up to date that thoroughly defined different Class III malocclusion phenotypes in Yemeni adults. Moreover, this is the first study that aimed to use the three-dimensional radiograph, instead of conventional cephalometric radiograph, to identify Class III malocclusion phenotypes.

## MATERIAL AND METHODS

### STUDY SAMPLE

A retrospective cross-sectional study was designed, and the sample was collected from patients’ orthodontic records in the Orthodontics department of Sana’a University, using their pretreatment CBCT. This study was designed following the STROBE guidelines, and conducted in adherence to the Declaration of Helsinki. The study protocol was approved by the Ethical Committee of the Faculty of Medicine and Health Sciences, Sana’a University, Yemen (Approval no.: ECA/SU/203). The pretreatment CBCT images were obtained from the archives of the Orthodontics department (Sana’a University, Yemen), and were collected and studied from April 2020 to December 2022. The sample size has been estimated based on data available from previous studies.^11^ ^,^ ^16^ The sample size was calculated with a confidence level (1 − α) of 95%, statistical power of 90%, precision (d) of 0.30, and variance (S^2^) of the quantitative variable of reference group equal to 0.69. The sample was calculated as 120; however, from the total 1,000 CBCTs, unfortunately only 80 CBCT images met the inclusion criteria. All scans were taken for standard orthodontic/orthognathic diagnosis and treatment planning purposes.

The inclusion criteria of the selected sample were: molar or canine Class III malocclusion; Wits appraisal < −0.5 and/or ANB ≤ 0; postpubertal subjects (16-35 years of age) who were in phase IV or V of the cervical vertebral maturation stage (CVMS), and had practically completed their growth - to ensure that the developing malocclusion phenotype was fully expressed. The exclusion criteria were: low quality data, patients with any type of syndrome or asymmetry, and patients with history of orthodontics treatment or craniofacial trauma.

### THREE-DIMENSIONAL ANALYSIS

**Figure 1:**
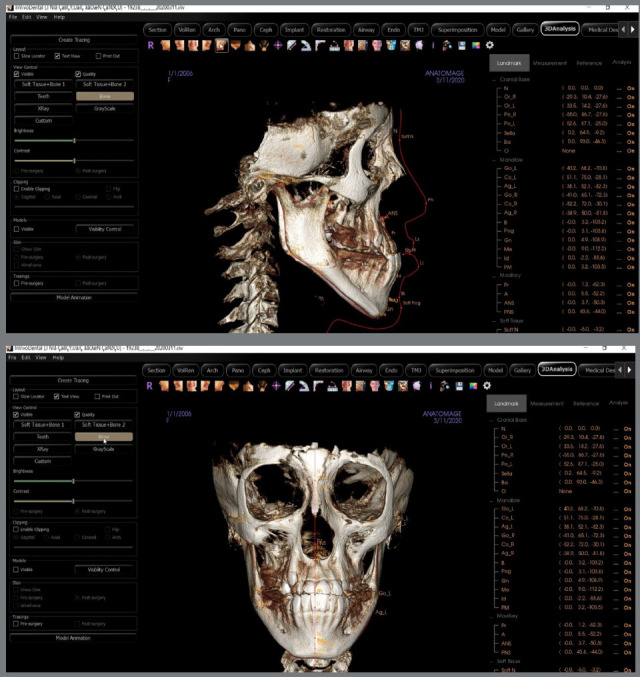
Three-dimensional anatomical landmarks.

CBCT was taken using a PaX-Flex3D P2 machine (ver. 1.0.0, Vatech, Korea, serial number 055,001482). The following parameters were used for the acquisition: field of view ≥8×8cm; 50-99 kv; 4-16MAs; scanning time of 15.0s; and the image voxel size equal to 0.2-0.3 mm. CBCT image has acquired with the subjects in a standard upright position, and Frankfort horizontal plane was parallel to the floor, guided by the crossing laser guide and front mirror. According to the imaging protocol, the CBCT imaging was done while patients were in centric occlusion and instructed not to swallow or move during the scanning process. The data were extracted in DICOM format (Digital Imaging and Communications in Medicine). Seventy-four linear, angular, and ratios measurements were three-dimensionally analyzed using Invivo v.6.0 software (Anatomage, Santa Clara, CA, USA). The three-dimensional definitions of the anatomical landmarks used in this study are presented in Table 1 and Figure 1. The measurements were applied to evaluate the required variables (46 skeletal, 18 dentoalveolar, and 10 soft tissues). These landmarks represent comprehensive craniofacial data and their relationships with each other. The identification of landmarks was viewed and confirmed on the three-dimensional planes. For all bilateral landmarks, the midpoint was considered rather than averaging the values of both sides, to make the measurements more accurate and valid. After identifying the anatomical landmarks, the Invivo 6.0 software automatically drew planes and performed all measurements (Table 1, Fig. 1). 

**Table 1:** Three-dimensional anatomical landmarks used in the study.

No	Name	Abbreviation	Definition
1	Nasion	N	The most anterior and midpoint of the fronto-nasal suture
2	Sella	S	The geometric center of pituitary fossa in the middle cranial fossa, in sagittal and axial views
3	Basion	Ba	The most lower point on the anterior-inferior margin of foramen magnum in the midline of skull base
4	Incisive foramen	IF	The center of incisive foramen center mediolateral, posterior to the central incisors at maxillary mid palatine
5	Right/left orbitale	Or-R/L	The right or left most inferior and middle point on the infraorbital rim of the maxilla
6	Right/left porion	Po-R/L	The right or left most outer and superior bony points of the external acoustic meatus
7	Subspinale	A	The deepest innermost and middle point on the contour of the premaxilla
8	Supramentale	B	The deepest innermost and middle point on the contour of the mandible
9	Gnathion	Gn	The most anteroinferior and middle point of the mandibular symphysis
10	Right/left gonion	Go-R/L	The right or left midpoint at angle of the mandible, halfway between the corpus and the ramus
11	Menton	Me	The most inferior and middle point of the chin on the outline of the mandibular symphysis
12	Pogonion	Pog	The most anterior and middle point on mandibular symphysis
13	Anterior nasal spine	ANS	The most anterior and middle point of the anterior nasal spine of the maxilla
14	Posterior nasal spine	PNS	The most posterior and middle point of the posterior nasal spine of the palatine bone
15	Infradentale	Id	The most anterosuperior and middle point on mandibular alveolus
16	Right/left condylion	Co -R/L	The midpoint of the right and left most posterosuperior point on mandibular condyle
17	Posterior condylar point	CP-R/L	The midpoint of the right and left most posterior point of the condylar head
18	Upper first molar	U6-R/L	The midpoint of the most superior point of the mesial buccal cusp of the right and left maxillary first molar
19	Lower first molar	L6-R/L	The midpoint of the most superior point of the mesial buccal cusp of the right and left mandibular first molar
20	Upper incisor	U1-R/L	Constructed between the midpoint of the incisal edge and the most superior and middle point of the root apex of the most protruded right or left maxillary central incisor
21	Lower incisor	L1-R/L	Constructed between the midpoint of the incisal edge and the most superior and middle point of the root apex of the most protruded right or left mandibular central incisor
22	Upper labrale superius	Uls	The most anterior and middle point on the muco-cutaneous junction of upper lip and philtrum
23	Lower labrale inferius	Lls	The most anterior and middle point on muco-cutaneous border of the lower lip
24	Pronasale	Pn	The most prominent and middle point on the tip of the nose
25	Subnasale	Sn	The midpoint on the nasolabial soft tissue contour between the columella crest and the upper lip
26	Soft tissue nasion	STN	The midpoint of greatest concavity in the midline between the forehead and the nose
27	Soft tissue pogonion	STPog	The most prominent or anterior point and middle on the soft tissue chin in the midsagittal plane
28	Soft tissue menton	STMe	The most inferior and middle point on the contour of the soft tissue chin
29	Upper lip anterior point	UL	The most anterior and middle point of the upper lip
30	Lower lip anterior point	LL	The most anterior and middle point of the lower lip

### RELIABILITY ASSESSMENT

The ability to perform the analysis according to the required standard analysis protocol was verified before analyzing the data. To improve the measurement’s validity and accuracy, a six-months training has been taken with dental radiologist. The intra-examiner reliability of three-dimensional craniofacial measurements was tested, and 20% randomly chosen data were retraced and digitized three times at three weeks’ interval, by the main operator (FB). The method error between the replicated tracing was calculated using the intraclass correlation and Cronbach’s alpha tests. There was very good intra-observer agreement regarding all measurements, with Cronbach’s alpha reliability coefficients ranging from 0.754 to 0.995.

### STATISTICAL ANALYSIS

Data was analyzed by using the IBM SPSS Statistics version 26.0 software. Due to the large number of variables, multivariate analysis such as PCA and CA were selected. These statistical tests aimed to determine the most homogeneous groups of individuals with various Class III phenotypes.

Firstly, the data was reduced by PCA, to dimensionally reduce a large set of variables into a smaller one that still contains most of the information of the large set prior to CA. The smaller set of new variables is known as components: 74 components were arranged in descending order by Eigenvalues - as the component order increases, less data is collected by the components. For components assessment, the Varimax rotation (Kaiser normalization) was used to transform the initial factors into new informative ones that are easier to interpret.

Secondly, a partitioned CA of extracted principal components (PCs) was performed, with methods based on the K-means algorithms. Finding the optimal number of clusters is an essential part of this algorithm. A commonly used method for finding the optimal K-value is the Elbow method, which involves plotting the variance, explained by different number of clusters, and identifying the Elbow point, where the rate of variation drops sharply, suggesting an appropriate cluster count for analysis. Means and standard variations (SD) for the variables in each cluster were calculated, and one-way ANOVA was used to detect differences between groups. The two-way cluster comparisons were performed. For comparisons between genders in each cluster, an independent t-test was used. P-values < 0.05 were considered statistically significant. 

## RESULTS

**Figure 2:**
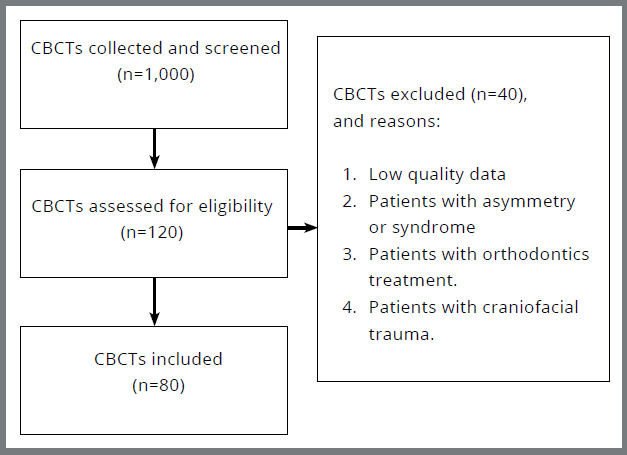
Flowchart of sample selection.

Initially, 1,000 CBCTs from patients’ files were collected and screened. Only 120 CBCTs were assessed for eligibility and, from this group, 40 CBCTs were excluded (n=40), according to exclusion criteria. Therefore, the final sample was 80 CBCTs. The sample consisted of 80 Yemeni adults (46 males, 66.7%, 34 females, 33.3%) with the mean age of 22.61 ± 5.13 years. This study used 74 variables; moreover, the results of the PCA identified 8 PCs that explained 78.9% of the overall variation in the data. The 8 PCs have been selected as they represented the greatest diversity in the collected data, and were obvious in their anatomic explanations (Fig. 2, Table 2). 

**Table 2:** Summary of principal components (PC) analysis.

Principal components	PC1	PC2	PC3	PC4	PC5	PC6	PC7	PC8
Variance explained^a^ (%)	31.581	8.484	8.483	7.510	6.779	6.486	5.487	4.104
Cumulative percentage^b^ (%)	31.581	40.065	48.548	56.058	62.837	69.323	74.811	78.914
Variables^c^	Max. length (ANS-PNS)(mm)	FMIA (degrees)	Post. face height (mm)	FMA (degrees)	LPDH (mm)	Facial taper (N-Gn-Go) (degrees)	SNB (degrees)	ANB (degrees)
	Ant. cranial base (SN) (mm)	L1-NB (degrees)	Ant. face height (mm)	Y (growth)-axis (degrees)	UPDH (mm)	SN- Pog (degrees)	SNA (degrees)	OJ (mm)
	Man. unit length (Co-Gn) (mm)	U1-L1 (degrees)	PFH:AFH (%)	Upper lip to ST N (mm)	UADH (mm)	L1-Apog (degrees)	OP-SN	Wits appraisal
	Mid face (Co-A) (mm)	U1-FH (degrees)	A-N perp FH (mm)	B-N perp FH (mm)	N-B//Hp (mm)	Chin angle Id-pog-MP (degrees)	AB plane (degrees)	Angle of convexity (NA-Apog) (degrees)
	Man. base length (Go-Gn) (mm)	U1-NA (degrees)	Lower face height (mm)	Max. Man Difference	N-Pg//Hp (mm)	SN-Ba (degrees)	SN-GoGn (degrees)	Op-FH (degrees)
	Up face height (N-ANS) (mm)	L1-NB (mm)	LFH/UFH (%)	Lower lip to ST N (mm)		Facial angle (degrees)(FH-NPg)		
	Ramus height (Co-Go) (mm)	Overbite (mm)	Gonial (degrees)(Co-Go-Me)					

^a^ represents the variables explained by each principal component in PCA, ^b^ shows the cumulative variables explained by each added PC sequentially. ^c^ displays the variables contributing the most in each PC.

C1 was the most observed cluster (n=26, male=36.4%, female=63.6%). This cluster was characterized by short anterior and posterior cranial bases, moderate maxillary retrusion, slightly normal mandibular plane, moderate mandibular protrusion, slightly protrusive chin, slightly decreased mandibular size, increased lower facial height (LFH) and total facial height (TFH), decreased upper facial height (UFH), dentoalveolar maxillary proclination and mandibular retroclination, protrusive upper and lower lips, and average facial type. 

C2 (n=21, male=63.2%, female=36.8%) was characterized by short anterior and posterior cranial bases, slight maxillary retrusion, steep mandibular plane, slight mandibular protrusion, slightly protrusive chin, increased mandibular size, increased LFH and TFH, decreased UFH, proclined maxillary and retroclined mandibular teeth, protrusive upper and lower lips, hyperdivergent facial type. 

C3 (n=21, male=73.7%, female=26.3%) was characterized by short anterior and posterior cranial bases, maxilla within the normal range, flat mandibular plane, severe mandibular protrusion, protrusive chin, increased mandibular size, long ramus, decreased LFH and TFH, increased UFH, nearly normal maxilla, and lingually inclined mandibular teeth, normal upper lip, protrusive lower lip, concave profile, hypodivergent facial type.

**Figure 3:**
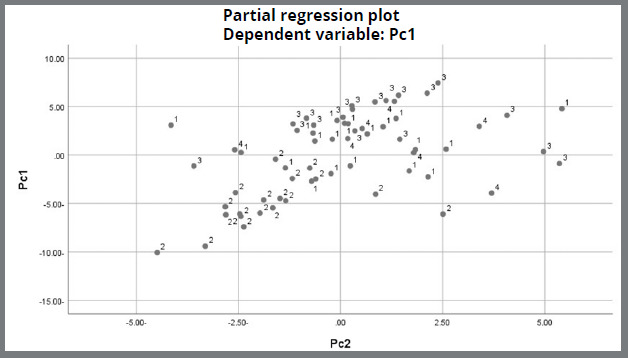
Scree plot of Class III clusters.

**Figure 4:**
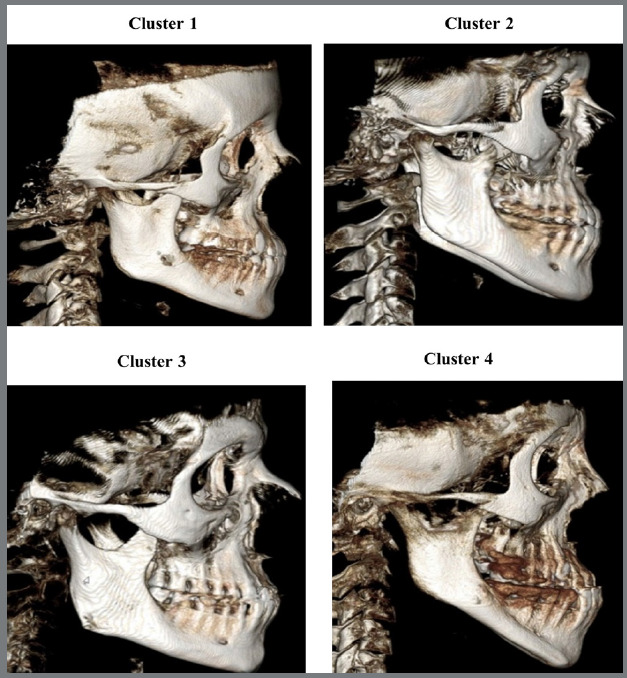
C1 (moderate phenotype), C2 (borderline phenotype), C3 (mandibular protrusion phenotype), C4 (severe phenotype).

C4 (n=12, male=57.1%, female=42.9%) was the least observed cluster, and was characterized by short anterior and posterior cranial bases, maxillary deficiency, increased mandibular plane, severe mandibular protrusion, protrusive chin, decreased total mandibular size, short ramus, increased LFH and TFH, decreased UFH, proclined maxillary and retroclined mandibular teeth, retrusive upper lip, more protrusive lower lip, hyperdivergent facial type (Fig. 3 and 4, Table 3).

**Table 3:** Mean and standard deviation (SD) for each cluster and one-way analysis of variance (ANOVA) between clusters.

Cephalometric measurements	Cluster 1 n=26		Cluster 2 n=21		Cluster 3 n=21		Cluster 4 n=12		Sig
	Mean	SD	Mean	SD	Mean	SD	Mean	SD	
SN-Ba	131.3	11.2	130.16	7.81	123.04	5.68	128.57	7.75	***
Ant. cranial base	65.26	3.2	59.50	2.0	72.37	1.6	67.76	5.8	***
Post. cranial base	34.81	8.4	40.35	10.8	27.41	3.9	30.74	4.5	***
SNA	75.43	5.4	77.72	4.2	74.40	4.8	83.91	5.0	***
NA-APog	173.3	1.5	177.1	1.7	167.0	6.5	173.5	2.1	***
Co-ANS	72.14	5.6	85.32	4.5	68.56	8.0	68.36	6.2	***
N-A //HP	2.94	1.0	4.96	2.5	4.86	0.7	3.14	1.5	***
A-N perp FH	56.48	4.1	54.79	2.7	51.27	6.2	54.59	5.2	**
ANS-PNS	53.78	8.8	45.12	6.0	46.89	2.0	43.44	3.5	***
SNB	78.72	5.3	78.38	3.6	81.45	3.8	86.63	4.8	***
FH-NP	7.23	2.4	3.69	4.0	13.14	2.7	9.83	2.4	***
Id-Pog-MP	70.90	4.64	68.73	5.54	80.42	4.32	76.89	4.07	***
Go-Pog	73.45	4.5	78.26	3.2	85.04	4.7	75.61	2.9	***
N-Gn-Go	75.36	14.5	83.37	4.4	64.95	5.3	82.43	22.8	***
N-B//Hp	5.68	0.6	8.53	6.19	4.17	3.2	5.66	4.30	***
N-Pg//Hp	3.27	1.6	7.68	3.2	1.70	0.7	3.94	2.7	***
B-Nperp FH	94.31	3.3	89.49	3.6	103.3	14.9	91.29	9.2	***
Pg-Nperp FH	4.76	3.7	13.05	2.31	3.58	2.0	5.68	3.2	***
Pg-NB	0.88	0.8	.72	0.4	1.60	1.4	.90	0.8	*
Co-Gn	100.1	7.9	120.1	12.5	119.3	12.7	116.89	6.7	**
Co-Go	53.59	3.5	59.67	8.5	47.75	4.8	51.80	3.8	***
Go-Gn	68.53	5.7	78.60	5.1	80.58	2.6	70.34	9.7	***
Co-Go-Me	123.5	6.0	110.5	7.6	127.5	11.4	128.1	8.1	***
Sella-Ar-Go	165.0	3.4	172.2	2.8	154.2	10.2	163.1	4.8	***
ANB	-2.91	0.5	-1.01	0.6	-5.96	3.3	-3.23	1.3	***
AB plane angle	3.03	0.6	.84	0.8	5.19	2.4	4.10	1.8	***
SN-Pog angle	82.38	4.6	74.05	2.8	105.4	31.9	86.71	8.7	***
Mid face length	90.35	5.1	94.10	3.8	92.39	4.7	90.59	8.0	NS
Max. Man differential	29.91	3.1	25.50	3.0	37.44	8.9	27.64	2.3	***
Wits appraisal	-2.86	0.5	-1.20	0.9	-5.96	3.3	-2.92	1.6	***
Y growth-axis	56.82	5.2	54.67	1.7	54.24	2.57	52.93	3.2	***
Y-axis	69.31	7.58	67.15	5.93	65.82	5.9	64.54	3.1	***
IMPA	85.54	3.2	94.74	3.8	76.43	2.5	84.96	7.0	***
Ant. face height	63.20	8.2	67.35	7.0	66.21	8.5	58.00	2.8	*
Post. face height	41.18	6.4	46.98	6.2	51.26	1.5	37.30	2.3	***
Upper face height	48.74	3.5	40.12	4.6	60.63	3.9	49.14	8.4	***
Lower face height	64.45	3.4	66.16	6.9	53.37	4.1	71.32	6.6	***
MP-SN	35.33	9.6	35.53	4.7	44.32	1.6	43.48	2.9	***
PFH:AFH	51.82	6.1	55.58	0.0	60.21	0.0	53.43	0.0	***
Nasal height	55.16	0.04	54.08	0.04	50.03	0.04	52.68	0.03	***
LFH/TFH	71.33	0.05	70.03	0.06	66.39	0.05	69.34	0.04	***
Face height	90.82	3.7	86.36	2.31	90.88	3.17	88.08	2.43	***
OP-SN	18.69	5.2	16.39	3.2	17.26	2.6	16.40	7.8	NS
SN-GoGn	35.88	4.3	25.28	2.7	44.31	3.2	37.14	7.3	***
FH-SN	12.59	2.6	13.89	1.6	17.63	1.0	15.14	2.0	***
FMA	20.76	7.1	24.06	4.0	33.79	2.3	23.20	8.3	***
Op-FH	9.23	3.3	6.77	2.68	8.50	3.0	11.66	5.2	***
U1-SN	103.8	7.9	95.19	4.9	112.3	3.4	99.84	9.4	***
U1-NA (mm)	16.12	9.3	15.91	2.7	32.95	2.0	25.84	4.6	***
U1-NA angle	26.16	4.8	19.45	3.5	34.18	2.3	29.40	8.3	***
U1- Palatal plane angle	118.18	6.97	118.57	1.15	114.95	7.04	109.58	4.57	***
L1-NB angle	20.34	5.8	25.26	3.8	15.36	7.3	20.29	4.3	***
L1-NB (mm)	3.76	0.6	5.50	0.5	6.72	0.5	4.76	1.5	***
L1-Apog angle	25.35	4.3	21.91	2.8	26.97	0.8	24.42	4.5	***
L1-Apog (mm)	4.83	1.2	5.64	0.6	7.40	2.3	5.38	2.0	***
FMIA	65.75	4.7	64.37	5.6	75.31	10.3	71.73	5.9	***
U1-L1 angle	136.6	4.7	130.7	4.9	145.5	13.0	141.4	10.2	***
Overjet	-2.55	0.6	-1.05	0.2	-4.00	.000a	-3.00	1.0	***
Overbite	1.60	0.6	.48	0.3	3.63	1.3	2.23	1.2	***
UADH (mm)	109.5	2.3	103.5	1.0	116.4	1.8	111.7	4.5	***
UPDH (mm)	23.87	4.9	24.74	2.6	25.68	5.3	21.86	1.3	NS
LADH (mm)	76.06	5.2	71.71	1.5	78.81	2.4	80.91	5.8	***
LPDH	24.38	5.0	21.70	5.9	20.15	5.2	15.98	7.2	***
U1FH	107.0	2.7	102.3	2.9	113.5	1.9	109.4	4.1	***
Lower lip to S-plane	-8.51	8.8	-9.43	2.1	-3.78	1.2	-5.89	2.5	**
Upper lip to S-plane	-6.18	0.9	-4.15	0.7	-10.2	1.0	-7.67	2.8	***
Upper lip - ST N perp FH	14.84	5.74	19.66	6.89	13.46	5.89	15.39	5.16	***
Lower lip - ST N perp FH	14.99	4.99	14.89	5.68	13.15	4.65	14.51	5.32	***
ST pog to ST N perp FH	14.13	4.71	12.25	6.20	15.23	5.12	12.14	4.64	***
Upper lip length (mm)	11.77	2.61	9.75	3.37	8.76	3.00	8.93	3.23	***
Lower lip length (mm)	29.64	4.3	29.42	4.1	25.63	0.7	30.29	4.2	***
UL-TV (mm)	2.02	1.3	3.16	0.02	2.08	1.48	1.83	1.01	***
Ll-TV (mm)	2.35	1. 74	2.23	2.09	2.60	1.00	1.94	1.76	***
Facial angle	177.1	1.7	174.7	0.6	172.5	1.2	167.4	6.3	***

NS = not significant. *** Significant mean difference.

## DISCUSSION

Skeletal Class III malocclusion has its own specific characteristics, which derive from genetic process. The identification and detailed description of phenotypic subtypes in the target population may allow one to obtain accurate objective criteria for the development of future studies, analyzing responses in the treatment, and analyzing etiological and differentiating factors of each sub-phenotype. In this study, three-dimensional analysis of skeletal, dentoalveolar, and soft tissue measurements have been performed to characterize Class III malocclusion sub-phenotypes by PCA and CA. The skeletal classification of samples has been determined using the ANB and Wits appraisal. The PCA of this study revealed eight axis models, which was similar to the results of the three-dimensional study done by Al-Shoaibi et al.^11^ When comparing this results with two-dimensional studies, the numbers of the axis models revealed two, seven, and ten components that covered 84%, 93.4%, and 92.9%, respectively.^14^ ^,^ ^17^ ^,^ ^18^ This is attributed to the inclusion of large numbers of skeletal variables to construct their axis models, while excluding other variables such as dental and soft tissue measurements. According to the PCA generated in this research, the first four axis models consisted mainly of sagittal, dental parameters, vertical variables, and anteroposterior relation of maxilla and mandible. 

Eight factors derived from PCA analysis were used as variables for CA to classify the skeletal Class III malocclusion. The number of clusters not only depends on ethnic groups,^19^ but also can be affected by the type of cluster that has been used, whether hierarchical,^20^ diffuse,^21^ mixed^17^ or K-mean, which have been used in previous studies^10^ ^,^ ^11^ ^,^ ^16^ and in the present study. Additionally, the present study classified Class III into four phenotypes, which was similar to Li et al.^10^ and Al-Shoaibi et al.^11^, as it revealed the most significant, meaningful and acceptable phenotypes. 

In this study, the characteristics of C1 were similar to those found in C1 by Al-Shoaibi et al.^11^ This might be due to skeletal Class III of mixed origin being the most common type of this malocclusion. In Al-Shoaibi et al.^11^ study, the group with the lowest number of C2 subjects was characterized by severe deficiency and protrusion of mandible, which was equivalent to C4 in this study. This could be due to the severe combination of maxillary retrognathism and mandibular prognathism, which is the less frequently found in adults. In contrast, the models with slight retrusion of maxilla and mandible (C2), and near normal maxilla and severe mandibular protrusion (C3) were not reported by Al-Shoaibi et al.^11^ Interestingly, this could be due to the different ethnic origin of the sample population.

Overall, this study showed different clusters description of skeletal Class III malocclusion regarding vertical, sagittal, dental, and soft tissue, compared to the clusters reported by Al-Shoaibi et al.^11^ This might be due to the dentofacial profile of Yemeni population, which has particular characteristics when compared to Chinese population. Moreover, this suggests that race should be considered when diagnosing facial structures.

Regarding the gender dimorphism, this study showed several linear measurements that were higher in males than female patients. However, in angular measurements, the majority of measurements showed no statistically significant difference between both genders. This finding was similar to Al-Shoaibi et al.^11^

This study was the first one in Yemen that used multivariate analysis in three-dimensional CBCT. The clusters description of this study has been compared only to another three-dimensional study, because both studies defined comprehensive craniofacial parameter of Class III skeletal malocclusion, which is limited by conventional two-dimensional approaches. Limitations of the study included the small sample size, due to the lack of available data. Another limitation of this study was the start date, during the COVID-19 pandemic, which reduced data collection during this period. Future studies with larger samples are required to understand the differences between regional and environmental factors.

## CONCLUSION

This study identified four phenotypes of skeletal Class III malocclusion, with eight PC accounting for 78.9% of the total variation in the data produced from 74 variables among Yemeni population. These included moderate phenotype, borderline Class III, Class III mandibular prognathism, and Class III severe combination of maxillary deficiency and mandibular prognathism. Skeletal Class III is mainly affected by genes, and the detection of these genes would help to predict growth changes and the possible treatment of this malocclusion. In order to understand this genetic component, it is necessary first to identify the various sub-phenotypes that are present within Class III skeletal malocclusion. Such classification is advantageous to provide differential diagnosis that lead to the definition of an accurate and effective treatment plan. Furthermore, it opens a world of possibilities for future genetic analysis, such as genotypic and linkage studies.

## Supplementary table 1: Three-dimensional craniofacial skeletal measurements used in the study.

[Table t1app]

**Table t1app:**

Measurement	Definition
Cranial base	
Cranial base angle (NS.Ba) (degrees)	The angle formed by nasion (N), sella (S) and basion (Ba)
Anterior cranial base (S-N) (mm)	The distance measured between points S and N
Posterior cranial base (S-Ba) (mm)	The distance measured between points S and Ba
Maxilla	
SNA (degrees)	The angle formed between the NA line and the SN plane
Angle of convexity (NA-APg) (degrees)	The angle formed by the intersection of the NA line and the APg line
N-A//HP (mm)	N perpendicular to A, parallel to HP. A perpendicular to HP is dropped from N, and the horizontal distance parallel to HP is measured from point A
A to N perp (FH) (mm)	The distance measured from point A to the nasion-perpendicular line to the FH plane
Maxillary unit length (Co-ANS) (mm)	The distance between the mid-condylar point and the anterior nasal spine (ANS)
Maxillary length (PNS-ANS) (mm)	The distance measured from the posterior nasal spine (PNS) and the anterior nasal spine (ANS)
Mandible	
SNB (degrees)	The angle formed between the NB line and the SN plane
Facial angle (FH-NPg) (degrees)	The angle formed by facial line [N-Pog] intersecting the FH
Gonial/mandibular angle (Co-Go-Me) (degrees)	The angle formed by line connecting condylion, gonion, and gnathion
Chin angle (Id-Pg-MP) (degrees)	The angle formed by infradental, pogonion, and the mandibular plane
Facial taper (N-Gn-Go) (degrees)	The angle formed by nasion, gnathion, and gonion
Mandibular unit length (Co-Gn) (mm)	The distance measured from the gnathion to condylion
Length of mandibular base (Go-Pg) (mm)	The distance measured from the gonion and the pogonion
Mandibular base length (Go- Gn) (mm)	The distance measured from the gonion and the gnathion
Ramus height (Co-Go) (mm)	The distance measured from the condylion and the gonion
N-B//HP (mm)	(N perpendicular to Pg, parallel to HP) Pg perpendicular to HP is dropped from N, and the horizontal distance parallel to HP is measured from point Pg
B to N perp (FH) (mm)	The distance measured from point B to the nasion-perpendicular line to the FH plane
Pg to N perp (FH) (mm)	The distance measured from pogonion (Pog) to the nasion-perpendicular line to the FH plane
Pg to NB (mm)	The distance measured perpendicular from pogonion to NB line
Post-facial Ht (Co-Gn) (mm)	The distance measured from condylion to gnathion
Intermaxillary	
ANB (degrees)	The angle formed by the intersection of the NA and NB lines
Facial plane to AB (AB-NPg) (degrees)	The angle formed between the AB line and the N-Pg line
Facial plane to SN (SN-NPg) (degrees)	The angle formed between the N-Pg line and the SN plane
Y growth-axis (S-Gn-FH) (degrees)	The angle formed by the intersection of a line from the sella turcica to gnathion with the FH
Y-axis (N-S-Gn) (degrees)	The angle is formed by the intersection between the S-Gn line and the SN plane
Frankfort mandibular plane (FMA) (FH-MP) (degrees)	The angle formed between Frankfort horizontal line and the mandibular plane
SN-GoGn (degrees)	The angle formed between GoGn and SN lines
Occlusal plane to SN (Op-SN) (degrees)	The angle formed between occlusal plane and SN line
Occlusal plane to FH (degrees)	The angle formed by the slope of the occlusal plane to the FH plane
FH-SN (degrees)	The angle formed by the Frankfort horizontal plane to SN line
Wits appraisal (AO-BO) (mm)	The distance measured along the occlusal plane from perpendicular points to A and B
Midface length (Co-A) (mm	The distance between the mid-condylar point and point A
Upper face height (N-ANS) (mm)	The distance measured from nasion (N) to anterior nasal spine (ANS)
Lower anterior facial height (LAFH) (ANS-Me) (mm)	The distance measured from the anterior nasal spine (ANS) to the menton (Me)
Anterior facial height (N-Me) (mm)	The distance measured from the nasion (N) to the menton (Me)
Posterior facial height (S-Go) (mm)	The distance between the mid-gonion point and the sella (S)
Maxillomandibular difference (Co-Gn-Co-A) (mm)	Effective mandibular length (Co-Gn) minus effective midface length (Co-A)
Face height (N-ANS/ANS-Me) (%)	The ratio of the upper anterior facial height (N-ANS) and lower anterior facial height (ANS-Me)
PFH: AFH (Co-Go/N-Me) (%)	The ratio of the posterior facial height (Co-Go) and anterior facial height (N-Me)
Dental	
U1-SN (degrees)	The angle formed between the long axis of upper incisor and SN line
U1-NA (degrees)	The angle formed between the axial inclination of maxillary incisors and NA lines
U1-NA (mm)	The distance measured between incisal tip of maxillary incisors and NA line
U1-FH (degrees)	The angle formed between long axis of upper incisor line and FH plane
U1-PP (degrees)	The angle formed between axial inclination of maxillary central incisor line and the palatal plane
L1-NB (degrees)	The angle formed between axial inclination of mandibular incisors and NB line
L1-NB (mm)	The distance measured between incisal tip of mandibular incisors and NB line
L1 protrusion (L1-APg) (degrees)	The angle formed between the long axis of the mandibular incisor and the A-Pg plane
L1 protrusion (L1-APg) (mm)	The distance measured from the incisal edge of the lower central incisor to A-Pg line
IMPA (L1-MP) (degrees)	The angle formed between axial inclination of mandibular incisor and the mandibular plane
Frankfort mandibular incisor angle (FMIA) (L1-FH) (degrees)	The angle formed between the Frankfort horizontal plane and the long axis of the mandibular incisor
Interincisal angle (U1-L1) (degrees)	The angle formed between axial inclination of maxillary and mandibular incisors
Upper anterior dental height (UADH) (U1-PP) (mm)	The perpendicular distance from the upper incisal edge projected to the palatal plane
Lower anterior dental height (LADH) (L1-MP) (mm)	The perpendicular distance from the lower incisal edge to the mandibular plane
Upper posterior dental height (UPDH) (U6-PP) (mm)	The perpendicular distance from the mesiobuccally cusp tip of the first upper molar to palatal plane
Lower posterior dental height (LPDH) (L6-MP) (mm)	The perpendicular distance from the mesiobuccally cusp tip of the first lower molar to the mandibular plane
Overjet (mm)	The distance measured the horizontal overlapping between labial surfaces of the upper and lower incisors
Overbite (mm)	The distance measured the vertical overlapping between the upper and lower incisor margins
Soft tissue	
Angle of facial convexity (degrees)	The angle formed by line connected nasion to subnasale to soft tissue Pogonion
Steiner’s S-line	The S-Shaped curved formed by the lower border of the nose and the soft tissue pogonion
Upper lip to S-plane (mm)	The distance from upper lip to Steiner’s S-line
Lower lip to S-plane (mm)	The distance from lower lip to Steiner’s S-line
Upper lip to ST. N perp (FH) (mm)	The distance measured from upper labrale superius to ST N-perpendicular FH
Lower lip to ST. N perp (FH) (mm)	The distance measured from lower labrale inferius to ST N-perpendicular FH
ST Pg to ST N perp (FH) (mm)	The distance measured from ST pogonion to ST N-perpendicular FH
Upper lip length (Sn-ULs) (mm)	The distance measured from subnasale (Sn) to the upper labrale superius (Uls)
Lower lip length (Me-LLi) (mm)	The distance measured from menton (Me) to the lower labrale inferius (LLi)
Upper lip anterior (ULA-TVL) (mm)	The horizontal distance from TVL to upper lip anterior point
Lower lip anterior (LLA-TVL) (mm)	The horizontal distance from TVL to lower lip anterior point

NS=not significant. *** Significant mean difference.

## Supplementary table 2: Mean and standard deviation (SD) for each cluster and one-way analysis of variance (ANOVA) between genders.

[Table t2app]

**Table t2app:**

Three-dimensional measurements	Male n=46		Female n=34		P-value	Sig
	Mean	SD	Mean	SD		
SN-Ba	138.84	5.97	130.55	5.50	0.000	***
Ant. cranial base	67.45	5.74	61.89	5.05	0.000	***
Post. cranial base	35.22	7.96	31.42	5.76	0.001	***
SNA	76.01	5.12	77.54	6.02	0.276	NS
NA-APog	171.7	6.72	173.8	2.14	0.073	NS
Co-ANS	74.64	9.62	74.24	8.94	0.862	NS
N-A //HP	4.56	1.94	3.45	1.46	0.009	**
A-N perp FH	54.62	5.39	53.94	4.38	0.570	NS
ANS-PNS	47.06	5.02	49.90	9.25	0.144	NS
SNB	80.01	4.50	80.51	5.67	0.699	NS
FH-NP	8.46	5.37	7.80	3.77	0.554	NS
Id-pog-MP	84.61	5.28	79.72	5.54	0.001	***
Go-Pog	83.30	5.19	79.84	3.43	0.002	**
N-Gn-Go	74.18	13.73	77.03	13.28	0.394	NS
N-B//Hp	4.45	4.16	4.67	2.55	0.797	NS
N-Pg//Hp	3.84	3.19	4.54	3.09	0.367	NS
B-Nperp FH	95.22	6.63	95.10	13.77	0.966	NS
Pg-Nperp FH	98.21	6.18	100.1	18.08	0.593	NS
Pg-NB	1.36	1.14	0.62	0.50	0.001	***
Co-Gn	105.5	11.25	102.2	10.86	0.217	NS
Co-Go	55.28	8.56	51.10	3.93	0.010	**
Go-Gn	71.45	9.03	69.16	8.32	0.285	NS
Co-Go-Me	110.4	11.92	122.8	9.52	0.000	***
Sella-Ar-Go	161.4	10.47	166.9	5.86	0.008	**
ANB	-3.73	3.28	-2.66	1.21	0.069	NS
AB plane angle	3.42	2.62	2.76	1.59	0.211	NS
SNPog angle	91.27	26.47	81.39	8.16	0.035	*
Mid face length	93.87	4.70	89.59	4.70	0.000	***
Max. Man Differential	31.02	8.91	29.95	3.37	0.497	NS
Wits appraisal	-3.64	3.32	-2.78	1.14	0.143	NS
Y growth-axis	54.95	2.60	56.62	4.44	0.077	NS
Y-axis	65.82	5.9	64.59	3.8	0.053	NS
IMPA	86.34	8.73	84.41	6.51	0.303	***
Ant. face height	67.55	7.50	60.93	7.06	0.000	***
Post. face height	45.34	6.27	39.44	5.29	0.000	***
Upper face height	53.59	3.5	48.21	7.61	0.000	***
Lower face height	62.08	9.90	71.73	4.1	0.000	***
MP-SN	39.85	6.98	37.39	7.84	0.187	NS
PFH:AFH	0.56	0.04	0.54	0.06	0.123	NS
LFH/TFH	72.38	5.94	69.35	4.7	0.000	***
Nasal height	53.59	3.5	52.93	3.2	0.000	***
Face height	92.78	3.4	88.08	2.43	0.000	***
OP-SN	18.21	4.08	16.31	4.71	0.089	NS
SN-GoGn	35.50	8.64	35.27	7.88	0.912	NS
FH-SN	15.87	2.22	13.07	2.63	0.000	***
FMA	25.79	7.92	25.45	7.13	0.854	NS
Op-FH	7.81	5.35	8.56	3.65	0.502	NS
U1-SN	103.9	9.36	102.6	8.60	0.534	NS
U1-NA mm	24.38	8.90	18.61	9.40	0.014	**
U1-NA angle	27.92	7.28	25.49	6.73	0.162	NS
U1-Palatal plane angle	119.1	6.6	120.27	2.18	0.000	***
L1-NB angle	19.74	7.02	21.07	6.30	0.418	NS
L1-NB	5.51	1.30	4.83	1.35	0.043	*
L1-Apog angle	25.47	3.36	23.77	4.03	0.073	NS
L1-Apog	5.97	1.96	5.69	1.76	0.543	NS
FMIA	69.69	9.93	67.39	5.56	0.234	NS
U1-L1	138.9	11.45	136.7	8.08	0.368	NS
Overjet	-2.76	1.28	-2.34	1.10	0.157	NS
Overbite	2.29	1.67	1.44	1.00	0.012	**
UADH	110.9	5.63	108.7	4.89	0.093	NS
UPDH	25.50	4.70	23.01	3.39	0.014	**
LADH	76.01	4.90	76.25	4.93	0.840	NS
LPDH	31.97	4.07	30.72	3.38	0.175	NS
U1FH	108.9	4.96	106.3	4.91	0.038	*
Lower lip to S-plane	-5.98	3.08	-8.70	7.69	0.080	NS
Upper lip to S-plane	-7.53	2.78	-6.07	2.20	0.020	*
Upper lip to ST N perp FH	19.70	6.91	18.90	5.79	0.663	NS
Lower lip-ST N perp FH	14.89	5.68	14.51	5.32	0.229	NS
ST pog to ST N perp FH	12.29	6.24	12.14	4.64	0.902	NS
Upper lip length mm	3.14	1.5	2.94	1.0	0.000	***
Lower lip length mm	2.23	2.09	2.08	1.48	0.000	***
UL-TV	2.09	1.48	1.85	1.03	0.000	***
LL-TV	2.64	1.04	1.99	1.47	0.000	***
Facial angle	175.7	0.7	173.7	2.3	0.009	**

NS=not significant. *** Significant mean difference.
